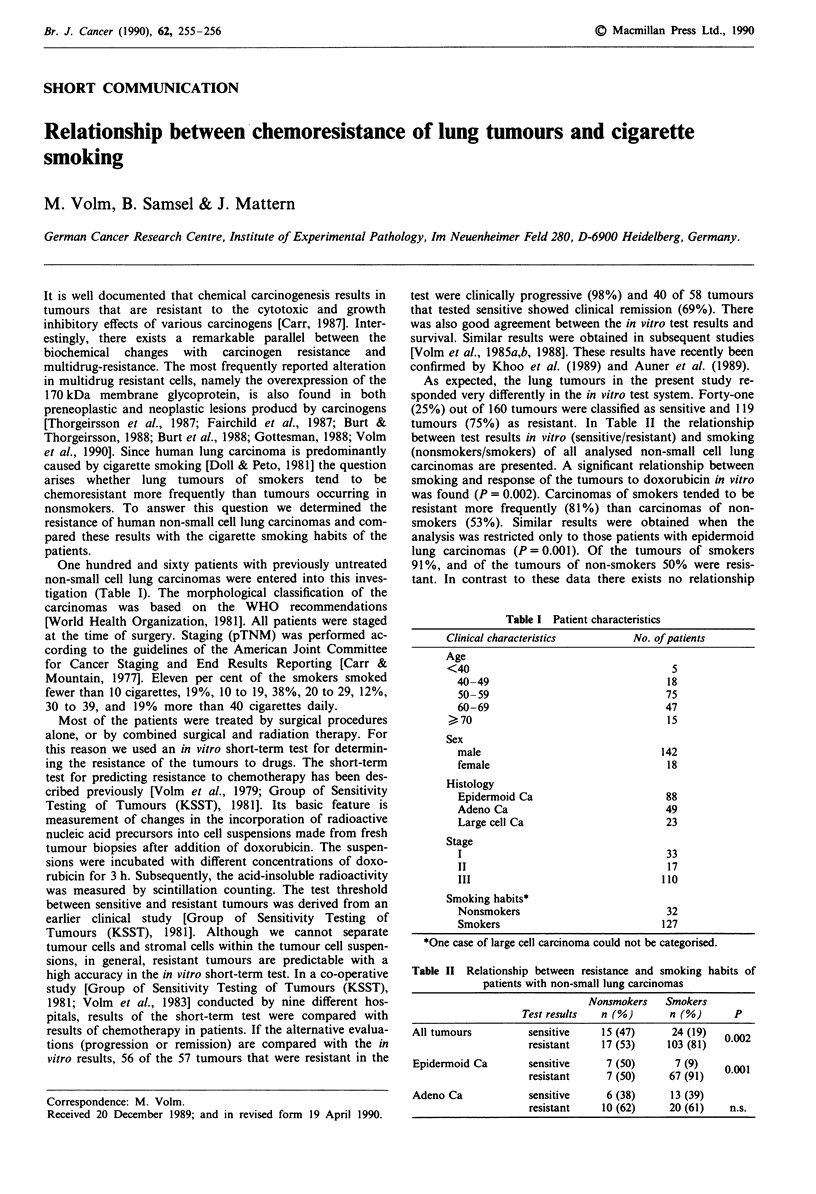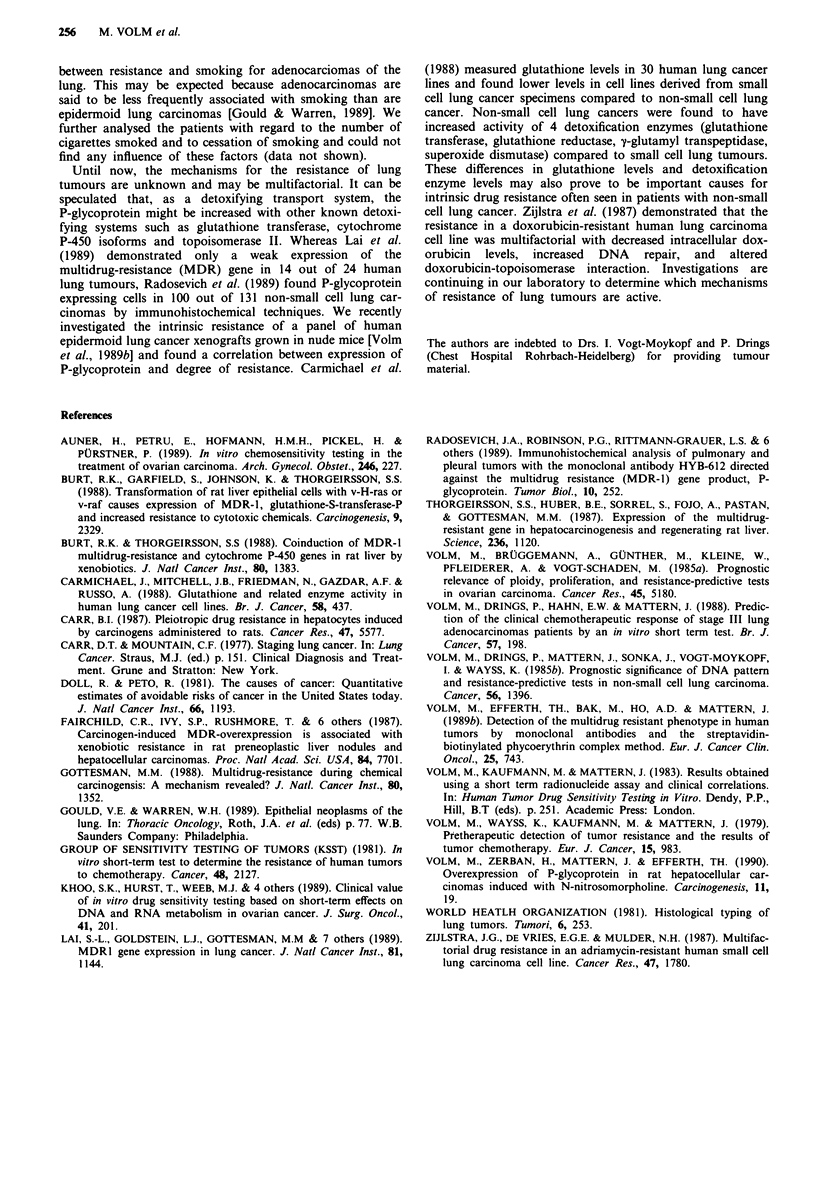# Relationship between chemoresistance of lung tumours and cigarette smoking.

**DOI:** 10.1038/bjc.1990.271

**Published:** 1990-08

**Authors:** M. Volm, B. Samsel, J. Mattern

**Affiliations:** German Cancer Research Centre, Institute of Experimental Pathology, Heidelberg.


Br. J. Cancer (1990), 62, 255-256 © Macmillan Press Ltd., 1990

SHORT COMMUNICATION

### Relationship between chemoresistance of lung tumours and cigarette smoking

M. Volm, B. Samsel & J. Mattern

*German Cancer Research Centre, Institute of Experimental Pathology, Im Neuenheimer Feld 280, D-6900 Heidelberg, Germany.*

It is well documented that chemical carcinogenesis results in tumours that are resistant to the cytotoxic and growth inhibitory effects of various carcinogens [Carr, 1987]. Interestingly, there exists a remarkable parallel between the biochemical changes with carcinogen resistance and multidrug-resistance. The most frequently reported alteration in multidrug resistant cells, namely the overexpression of the 170 kDa membrane glycoprotein, is also found in both preneoplastic and neoplastic lesions producd by carcinogens [Thorgeirsson *et al.*, 1987; Fairchild *et al.*, 1987; Burt & Thorgeirsson, 1988; Burt *et al.*, 1988; Gottesman, 1988; Volm *et al.*, 1990]. Since human lung carcinoma is predominantly caused by cigarette smoking [Doll & Peto, 1981] the question arises whether lung tumours of smokers tend to be chemoresistant more frequently than tumours occurring in nonsmokers. To answer this question we determined the resistance of human non-small cell lung carcinomas and compared these results with the cigarette smoking habits of the patients.

One hundred and sixty patients with previously untreated non-small cell lung carcinomas were entered into this investigation (Table I). The morphological classification of the carcinomas was based on the WHO recommendations [World Health Organization, 1981]. All patients were staged at the time of surgery. Staging (pTNM) was performed according to the guidelines of the American Joint Committee for Cancer Staging and End Results Reporting [Carr & Mountain, 1977]. Eleven per cent of the smokers smoked fewer than 10 cigarettes, 19%, 10 to 19, 38%, 20 to 29, 12%, 30 to 39, and 19% more than 40 cigarettes daily.

Most of the patients were treated by surgical procedures alone, or by combined surgical and radiation therapy. For this reason we used an *in vitro* short-term test for determining the resistance of the tumours to drugs. The short-term test for predicting resistance to chemotherapy has been described previously [Volm *et al.*, 1979; Group of Sensitivity Testing of Tumours (KSST), 1981]. Its basic feature is measurement of changes in the incorporation of radioactive nucleic acid precursors into cell suspensions made from fresh tumour biopsies after addition of doxorubicin. The suspensions were incubated with different concentrations of doxorubicin for 3 h. Subsequently, the acid-insoluble radioactivity was measured by scintillation counting. The test threshold between sensitive and resistant tumours was derived from an earlier clinical study [Group of Sensitivity Testing of Tumours (KSST), 1981]. Although we cannot separate tumour cells and stromal cells within the tumour cell suspensions, in general, resistant tumours are predictable with a high accuracy in the *in vitro* short-term test. In a co-operative study [Group of Sensitivity Testing of Tumours (KSST), 1981; Volm *et al.*, 1983] conducted by nine different hospitals, results of the short-term test were compared with results of chemotherapy in patients. If the alternative evaluations (progression or remission) are compared with the *in vitro* results, 56 of the 57 tumours that were resistant in the test were clinically progressive (98%) and 40 of 58 tumours that tested sensitive showed clinical remission (69%). There was also good agreement between the *in vitro* test results and survival. Similar results were obtained in subsequent studies [Volm *et al.*, 1985*a*,*b*, 1988]. These results have recently been confirmed by Khoo *et al.* (1989) and Auner *et al.* (1989).

As expected, the lung tumours in the present study responded very differently in the *in vitro* test system. Forty-one (25%) out of 160 tumours were classified as sensitive and 119 tumours (75%) as resistant. In Table II the relationship between test results *in vitro* (sensitive/resistant) and smoking (nonsmokers/smokers) of all analysed non-small cell lung carcinomas are presented. A significant relationship between smoking and response of the tumours to doxorubicin *in vitro* was found ($P = 0.002$). Carcinomas of smokers tended to be resistant more frequently (81%) than carcinomas of nonsmokers (53%). Similar results were obtained when the analysis was restricted only to those patients with epidermoid lung carcinomas ($P = 0.001$). Of the tumours of smokers 91%, and of the tumours of non-smokers 50% were resistant. In contrast to these data there exists no relationship

**Table I** Patient characteristics

| *Clinical characteristics* | *No. of patients* |
|---|---|
| Age | |
| <40 | 5 |
| 40-49 | 18 |
| 50-59 | 75 |
| 60-69 | 47 |
| ⩾70 | 15 |
| Sex | |
| male | 142 |
| female | 18 |
| Histology | |
| Epidermoid Ca | 88 |
| Adeno Ca | 49 |
| Large cell Ca | 23 |
| Stage | |
| I | 33 |
| II | 17 |
| III | 110 |
| Smoking habits* | |
| Nonsmokers | 32 |
| Smokers | 127 |

*One case of large cell carcinoma could not be categorised.

**Table II** Relationship between resistance and smoking habits of patients with non-small lung carcinomas

| | *Test results* | *Nonsmokers n (%)* | *Smokers n (%)* | *P* |
|---|---|---|---|---|
| All tumours | sensitive | 15 (47) | 24 (19) | 0.002 |
| | resistant | 17 (53) | 103 (81) | |
| Epidermoid Ca | sensitive | 7 (50) | 7 (9) | 0.001 |
| | resistant | 7 (50) | 67 (91) | |
| Adeno Ca | sensitive | 6 (38) | 13 (39) | |
| | resistant | 10 (62) | 20 (61) | n.s. |

Correspondence: M. Volm.
Received 20 December 1989; and in revised form 19 April 1990.

between resistance and smoking for adenocarciomas of the lung. This may be expected because adenocarcinomas are said to be less frequently associated with smoking than are epidermoid lung carcinomas [Gould & Warren, 1989]. We further analysed the patients with regard to the number of cigarettes smoked and to cessation of smoking and could not find any influence of these factors (data not shown).

Until now, the mechanisms for the resistance of lung tumours are unknown and may be multifactorial. It can be speculated that, as a detoxifying transport system, the P-glycoprotein might be increased with other known detoxifying systems such as glutathione transferase, cytochrome P-450 isoforms and topoisomerase II. Whereas Lai *et al.* (1989) demonstrated only a weak expression of the multidrug-resistance (MDR) gene in 14 out of 24 human lung tumours, Radosevich *et al.* (1989) found P-glycoprotein expressing cells in 100 out of 131 non-small cell lung carcinomas by immunohistochemical techniques. We recently investigated the intrinsic resistance of a panel of human epidermoid lung cancer xenografts grown in nude mice [Volm *et al.*, 1989*b*] and found a correlation between expression of P-glycoprotein and degree of resistance. Carmichael *et al.* (1988) measured glutathione levels in 30 human lung cancer lines and found lower levels in cell lines derived from small cell lung cancer specimens compared to non-small cell lung cancer. Non-small cell lung cancers were found to have increased activity of 4 detoxification enzymes (glutathione transferase, glutathione reductase, γ-glutamyl transpeptidase, superoxide dismutase) compared to small cell lung tumours. These differences in glutathione levels and detoxification enzyme levels may also prove to be important causes for intrinsic drug resistance often seen in patients with non-small cell lung cancer. Zijlstra *et al.* (1987) demonstrated that the resistance in a doxorubicin-resistant human lung carcinoma cell line was multifactorial with decreased intracellular doxorubicin levels, increased DNA repair, and altered doxorubicin-topoisomerase interaction. Investigations are continuing in our laboratory to determine which mechanisms of resistance of lung tumours are active.

The authors are indebted to Drs. I. Vogt-Moykopf and P. Drings (Chest Hospital Rohrbach-Heidelberg) for providing tumour material.

### References

AUNER, H., PETRU, E., HOFMANN, H.M.H., PICKEL, H. & PÜRSTNER, P. (1989). *In vitro* chemosensitivity testing in the treatment of ovarian carcinoma. *Arch. Gynecol. Obstet.*, **246**, 227.

BURT, R.K., GARFIELD, S., JOHNSON, K. & THORGEIRSSON, S.S. (1988). Transformation of rat liver epithelial cells with v-H-ras or v-raf causes expression of MDR-1, glutathione-S-transferase-P and increased resistance to cytotoxic chemicals. *Carcinogenesis*, **9**, 2329.

BURT, R.K. & THORGEIRSSON, S.S (1988). Coinduction of MDR-1 multidrug-resistance and cytochrome P-450 genes in rat liver by xenobiotics. *J. Natl Cancer Inst.*, **80**, 1383.

CARMICHAEL, J., MITCHELL, J.B., FRIEDMAN, N., GAZDAR, A.F. & RUSSO, A. (1988). Glutathione and related enzyme activity in human lung cancer cell lines. *Br. J. Cancer*, **58**, 437.

CARR, B.I. (1987). Pleiotropic drug resistance in hepatocytes induced by carcinogens administered to rats. *Cancer Res.*, **47**, 5577.

CARR, D.T. & MOUNTAIN, C.F. (1977). Staging lung cancer. In: *Lung Cancer*. Straus, M.J. (ed.) p. 151. Clinical Diagnosis and Treatment. Grune and Stratton: New York.

DOLL, R. & PETO, R. (1981). The causes of cancer: Quantitative estimates of avoidable risks of cancer in the United States today. *J. Natl Cancer Inst.*, **66**, 1193.

FAIRCHILD, C.R., IVY, S.P., RUSHMORE, T. & 6 others (1987). Carcinogen-induced MDR-overexpression is associated with xenobiotic resistance in rat preneoplastic liver nodules and hepatocellular carcinomas. *Proc. Natl Acad. Sci. USA*, **84**, 7701.

GOTTESMAN, M.M. (1988). Multidrug-resistance during chemical carcinogensis: A mechanism revealed? *J. Natl. Cancer Inst.*, **80**, 1352.

GOULD, V.E. & WARREN, W.H. (1989). Epithelial neoplasms of the lung. In: *Thoracic Oncology*, Roth, J.A. *et al.* (eds) p. 77. W.B. Saunders Company: Philadelphia.

GROUP OF SENSITIVITY TESTING OF TUMORS (KSST) (1981). *In vitro* short-term test to determine the resistance of human tumors to chemotherapy. *Cancer*, **48**, 2127.

KHOO, S.K., HURST, T., WEEB, M.J. & 4 others (1989). Clinical value of *in vitro* drug sensitivity testing based on short-term effects on DNA and RNA metabolism in ovarian cancer. *J. Surg. Oncol.*, **41**, 201.

LAI, S.-L., GOLDSTEIN, L.J., GOTTESMAN, M.M & 7 others (1989). MDR1 gene expression in lung cancer. *J. Natl Cancer Inst.*, **81**, 1144.

RADOSEVICH, J.A., ROBINSON, P.G., RITTMANN-GRAUER, L.S. & 6 others (1989). Immunohistochemical analysis of pulmonary and pleural tumors with the monoclonal antibody HYB-612 directed against the multidrug resistance (MDR-1) gene product, P-glycoprotein. *Tumor Biol.*, **10**, 252.

THORGEIRSSON, S.S., HUBER, B.E., SORREL, S., FOJO, A., PASTAN, & GOTTESMAN, M.M. (1987). Expression of the multidrug-resistant gene in hepatocarcinogenesis and regenerating rat liver. *Science*, **236**, 1120.

VOLM, M., BRÜGGEMANN, A., GÜNTHER, M., KLEINE, W., PFLEIDERER, A. & VOGT-SCHADEN, M. (1985*a*). Prognostic relevance of ploidy, proliferation, and resistance-predictive tests in ovarian carcinoma. *Cancer Res.*, **45**, 5180.

VOLM, M., DRINGS, P., HAHN, E.W. & MATTERN, J. (1988). Prediction of the clinical chemotherapeutic response of stage III lung adenocarcinomas patients by an *in vitro* short term test. *Br. J. Cancer*, **57**, 198.

VOLM, M., DRINGS, P., MATTERN, J., SONKA, J., VOGT-MOYKOPF, I. & WAYSS, K. (1985*b*). Prognostic significance of DNA pattern and resistance-predictive tests in non-small cell lung carcinoma. *Cancer*, **56**, 1396.

VOLM, M., EFFERTH, TH., BAK, M., HO, A.D. & MATTERN, J. (1989*b*). Detection of the multidrug resistant phenotype in human tumors by monoclonal antibodies and the streptavidin-biotinylated phycoerythrin complex method. *Eur. J. Cancer Clin. Oncol.*, **25**, 743.

VOLM, M., KAUFMANN, M. & MATTERN, J. (1983). Results obtained using a short term radionucleide assay and clinical correlations. In: *Human Tumor Drug Sensitivity Testing in Vitro*. Dendy, P.P., Hill, B.T (eds). p. 251. Academic Press: London.

VOLM, M., WAYSS, K., KAUFMANN, M. & MATTERN, J. (1979). Pretherapeutic detection of tumor resistance and the results of tumor chemotherapy. *Eur. J. Cancer*, **15**, 983.

VOLM, M., ZERBAN, H., MATTERN, J. & EFFERTH, TH. (1990). Overexpression of P-glycoprotein in rat hepatocellular carcinomas induced with N-nitrosomorpholine. *Carcinogenesis*, **11**, 19.

WORLD HEATLH ORGANIZATION (1981). Histological typing of lung tumors. *Tumori*, **6**, 253.

ZIJLSTRA, J.G., DE VRIES, E.G.E. & MULDER, N.H. (1987). Multifactorial drug resistance in an adriamycin-resistant human small cell lung carcinoma cell line. *Cancer Res.*, **47**, 1780.